# Colostrum Extracellular Vesicle Isolation, Characterization, and Function

**DOI:** 10.3390/biomedicines14071549

**Published:** 2026-07-10

**Authors:** Samia Akter, Nada Fayez, Mohit Kumar, Susmita Sil, Howard E. Gendelman

**Affiliations:** Department of Pharmacology and Experimental Neuroscience, University of Nebraska Medical Center, Omaha, NE 68106, USA

**Keywords:** colostrum, extracellular vesicles, pro-inflammatory cytokines, NLRP3

## Abstract

**Background**: Colostrum extracellular vesicles (C-EVs) are nanoscale, bioactive vesicles with therapeutic potential. The mechanisms of action include the control of cellular and tissue homeostasis. These make C-EVs a novel means to control inflammatory and cellular dysfunctions. However, a limitation for their broad use is the ease of C-EV isolation and in ensuring their stability. **Methods**: Standard ultracentrifugation and gradient techniques used for EV recovery were employed, which included ultracentrifugation. Exodus dual-frequency ultrasonic nanofiltration (UNF) was a comparator used to overcome standard limitations by recovering pure vesicles at high concentrations. Both systems were evaluated for their abilities to recover clinical-grade C-EVs with optimal vesicle structural integrity and intact biological functions. **Results**: This study affirms UNF C-EV recovery by demonstrating intact Alix, CD63, Tsg101, and Flotillin antigens. The EVs maintained an intact bilayer structure with sizes ranging from to 50–200 nm. Functional tests showed preservation of their anti-inflammatory activities by suppression of pro-inflammatory cytokines and the NLRP3 inflammasome, caspase 1, interleukin-1, and 18 and maintaining cellular homeostasis. Processing time, high yield, and functional responses controlled cellular function. **Conclusions**: These data support the notion that UNF C-EVs can be recovered safely, at high yields, and reproducibly for future clinical applications.

## 1. Introduction

“First milk,” known as colostrum, is a concentrated nutrient-rich breast milk that a mother produces during pregnancy and after giving birth [1,2]. Colostrum is rich source of protein, fat, carbohydrates, vitamins, minerals, growth factors, immunoglobulins, proline polypeptides, and antioxidants. Each supports neonatal development and immunity [3,4,5]. Bovine colostrum is a “super food,” with nearly twice the fat and four times the protein, 250 times immunoglobulins, vitamins B_2_, B_12_, E, and D, and minerals (calcium, copper, iron, zinc, magnesium, manganese, and phosphorus) compared with mature milk [6,7,8]. Colostrum is rich in bioactive oligosaccharides and probiotics, which benefit the gut microbiota [4]. Colostrum supplements are heavily marketed for gut health, immune support, and athletic performance. However, to date, there is scant evidence to support any broad nutritional claims. Nonetheless, colostrum can serve as a natural therapeutic agent with regenerative, wound-healing, anti-inflammatory, and immunomodulatory functions [9]. Bovine colostrum can reduce intestinal inflammation by regulating IL-8. This is achieved by affecting the epithelial pathogen immunity. There is also evidence of neuroprotective activity in rat models of cerebral ischemia. Reduced neuroinflammation is associated with lower levels of pro-inflammatory cytokines, such as IL-1β, IL-6, and TNF-α, and improved neurological outcomes [10].

Recent studies support these claims for colostrum extracellular vesicles (C-EVs) [11]. Extracellular vesicles (EVs) are active components of colostrum, that facilitate cell-to-cell communication by transferring bioactive cargo. These include proteins, lipids, and nucleic acids [12]. While EVs demonstrate significant therapeutic potential for intestinal and hepatic health, their bioactivities are the active colostrum. Similar to crude colostrum, these factors can reduce gut inflammation, protect against liver injury [13], and improve gut barrier function [14]. Notably, EVs can treat ulcerative colitis by suppressing inflammatory responses and restoring the regulatory T cell (Treg)/Th17 immune balance, including the control of TLR4/NF-κB and NLRP3 signaling [15]. Additional studies have reported that EVs protect against mastitis [16], steatohepatitis [17], oxidative stress, ferroptosis [18], and rheumatoid arthritis [19]. Our previous work supports these observations by demonstrating that C-EVs are both neuroprotective and anti-inflammatory when tested in a rodent model of Parkinson’s disease (PD) [11]. While preclinical research offers translational pathways, bulk EV isolation is limited by the recovery techniques used. These include size-exclusion chromatography and serial ultracentrifugation methods. Both methods provide pure exosome preparations but are often limited by low sample capacity and time [20]. Higher-yield approaches such as polymer precipitation (PEG) and tangential flow filtration are more efficient but yield lower purity and have time constraints [21,22]. Ultrasonic Nanofiltration (UNF) provides an ultrafast isolation alternative (Exodus) [23,24]. UNF employs an automated dual-membrane nanofiltration system with periodic negative pressure oscillations (NPOs) and double-coupled harmonic oscillations (HOs) [25]. Comparative EV isolation techniques have shown that all methods preserve the physical and functional properties of EVs. However, the best recovery was achieved using UNF, which showed an equivalent vesicle size distribution with improved recovery. Nanoparticle Tracking Analysis (NTA) showed that the UNF approach yielded optimal morphology when examined by transmission electron microscopy (TEM) and cryogenic electron microscopy (cryo-EM). The critical anti-inflammatory properties of EVs were preserved. Western blotting analysis of EV markers demonstrated the high purity of the isolated EVs. These findings demonstrate that the UNF system enables rapid isolation with short processing times while achieving high yields with minimal contamination by colostrum cell components.

## 2. Methods

### 2.1. EV Isolation

Bovine colostrum (from Organic Livestock farming) was obtained from the Oehlerking Farm (Elmwood, NE, USA) within 24 h postpartum, then transported to the laboratory for isolation and characterization. As a control, C-EVs were isolated using the conventional Opti-Prep method (OPT) [11]. Briefly, 50 mL of colostrum was centrifuged at 4700× *g* for 20 min at 4 °C to remove fat globules and debris, followed by centrifugation at 2000× *g* for 20 min at 4 °C to remove smaller fat globules. The supernatant was filtered through Whatman grade 1 filter paper. After filtration, an equal volume of 0.25 M EDTA (pH 7) was added, and the mixture was incubated on ice for 20 min to remove the casein. The pH was then adjusted to 4.6 using 6 N acetic acid to precipitate the casein. Next, the samples were ultracentrifuged at 65,000× *g* for 1.5 h, followed by filtration through grade 1 filter paper and 0.45- and 0.22-micron filters. Ten milliliters of the filtrate was diluted with phosphate-buffered saline (PBS) and centrifuged at 110,000× *g* for 2 h. The resulting pellets were resuspended in PBS. Gradient ultracentrifugation was performed using 40, 30, 20, 10, and 5% OptiPrep density gradients at 186,000× *g* for 18 h at 10 °C using an SW 41 Ti swinging bucket rotor (Beckman Coulter, Indianapolis, IN, USA). Each fraction was collected and washed with PBS at 110,000× *g* for 90 min at 4 °C. The resulting pellets were resuspended in 1X PBS.

### 2.2. Ultrasonic Nanofiltration

C-EV was isolated by using UNF as a comparator (Exodus system, Cambridge, MA, USA) [15]. Fifty milliliters of colostrum was centrifuged at 2000× *g* for 30 min at 4 °C to remove cells and debris. The supernatant pH was adjusted to 4.6 with 2 M HCl to reach the isoelectric point of κ-casein, and then incubated on ice for 10–15 min. The samples were further centrifuged 2- times at 10,000× *g* for 60 min, followed by 10,000× *g* for 30 min at 4 °C to remove any remaining casein. The clarified supernatant was subsequently diluted with PBS (1:5) and filtered through 0.45 and 0.22-micron filters. Finally, the processed sample was loaded into the Exodus system for EV isolation, according to the manufacturer’s protocol.

### 2.3. Nanoparticle Tracking

The concentration and size distribution of C-EVs were determined by NTA using a NanoSight NS300 at 25 °C. Nanoparticle tracking analysis [16,17,18] was performed using an NS300 Nanosight (Malvern Panalytical Ltd., Westborough, MA, USA) with a green 532 nm laser. NTA 3.4 Build 3.4.4 software was used to acquire and analyze all measurements. The system was set to the following specifications: frame rate, 24,9825 fps; Slider Gain, 219; Shutter Speed, 30.8 ms; Screen Gain, 1.8; Camera Level, 14; and Detection Limit, 5. The samples were diluted 1:1000 in phosphate-buffered saline (PBS) that was EV-free. The measurement chamber was flushed with 1 mL of PBS before the analysis and between samples to prevent any carryover. For each run, 500 µL of the sample was loaded and videos were recorded in triplicate (3 × 60 s). Independent experiments were performed in triplicate. The data were processed using the Nanosight NTA software version 3.4 (Malvern Panalytical Ltd., Westborough, MA, USA), and the C-EV concentration was expressed as EVs/mL.

### 2.4. Electron Microscopy

EVs were initially fixed using 2% glutaraldehyde and then deposited onto 200-mesh Formvar-coated copper grids for 5 min at room temperature. After incubation, the grids were immersed in uranyl acetate solution for negative staining. Excess stain was removed by rinsing with PBS, and the grids were air-dried at room temperature prior to imaging. Transmission electron microscopy was performed using a Hitachi H-7500 microscope (Hitachi, Tokyo, Japan) at an acceleration voltage of 200 kV. Cryo-electron microscopy analysis was conducted at the contract facilities of the Hormel Institute, University of Minnesota Medical Research Center, Austin, MN, USA.

### 2.5. Dynamic Light Scattering

C-EVs were diluted in PBS (1:1000) prior to characterization using dynamic light scattering (DLS). DLS measurements were performed in triplicate for each sample using a Zetasizer Nano (Malvern Panalytical Ltd., Westborough, MA, USA). The measurement parameters were set as follows: dispersant, (PBS); temperature, 25 °C; viscosity, 1 cP; material refractive index (polystyrene latex), 1.590; and absorption, 0.010.

### 2.6. Cell Culture

The microglial cell line (BV2 cells; Cat no.: NCL2110P153, Creative Biolabs neuroS, Shirley, NY, USA) was maintained in Dulbecco’s Modified Eagle Medium (DMEM) supplemented with 10% (*v*/*v*) heat-inactivated fetal bovine serum (FBS) and 1% penicillin/streptomycin at 37 °C in a humidified 5% CO_2_ atmosphere, using 75 cm^2^ culture flasks for routine maintenance. For different experiments, 2.0 × 10^5^ (Western blotting) or 1.0 × 10^5^ (qPCR) cells were seeded in 6-well or 24-well plates and allowed to adhere for 12 h, respectively. The experimental protocol involved treating cells with C-EVs (500 EVs/cell) for 22 h, followed by stimulation with 100 ng/mL lipopolysaccharide (LPS) to induce inflammation. For qPCR analysis, cells were incubated with LPS for 2 h prior to RNA extraction to evaluate mRNA expression levels. For Western blot analysis, cells were incubated with LPS for 24 h before protein extraction to assess protein expression.

### 2.7. Western Blot

After EV isolation, an equal number of EVs (1 × 10^10^ EVs) were mixed with 5X Laemmli buffer. The samples were incubated at 99 °C for 15 min. For cellular protein analysis, BV2 cells treated with EVs and LPS were lysed in RIPA buffer, sonicated, and centrifuged, and the total protein concentration was quantified using the Pierce BCA Protein Assay kit (23227, Thermo Fisher Scientific, Waltham, MA, USA). Samples were prepared and loaded at equal concentrations and electrophoresed under reducing conditions in 10–15% polyacrylamide gels. After electrophoresis, the proteins were transferred onto polyvinylidene fluoride (PVDF) membranes (Cat. No. IPVH00010 (Millipore Sigma, St. Louis, MO, USA) and blocked with 5% non-fat dry milk prepared in 1X Tris-buffered saline containing 0.1% Tween-20. Subsequently, the membranes were incubated overnight at 4 °C with primary antibodies. After primary antibody incubation, the membranes were treated with secondary antibodies for 1 h at RT. Chemiluminescence detection was performed using SuperSignal West Pico, Dura, Femto, and Atto substrates (Cat. Nos. 34580, 34076, 34096, A38556; Thermo Fisher Scientific, Waltham, MA, USA) using an iBright750 Imager (Thermo Fisher Scientific, Waltham, MA, USA). Images were quantified using the ImageJ Launcher software (v1.4.3.67, NIH, Bethesda, MD, USA), and fold changes in protein expression were normalized to β-actin as an internal control.

### 2.8. RNA Isolation

Total RNA was extracted using 500 µL TRIzol™ Reagent (Thermo Fisher Scientific, Waltham, MA, USA). Subsequently, 100 µL of chloroform was added, and the samples were vigorously shaken and incubated for 3 min at room temperature, followed by centrifugation at 12,000× *g* for 15 min at 4 °C to achieve phase separation. The upper aqueous phase containing RNA was carefully transferred to a new tube. RNA was precipitated by adding 250 µL of isopropanol and incubating at room temperature for 10 min. Samples were centrifuged at 12,000× *g* for 10 min at 4 °C to pellet the RNA. The RNA pellet was washed with 1 mL of 75% ethanol, vortexed briefly, and centrifuged at 7500× *g* for 4 min at 4 °C. The supernatant was discarded, and the pellet was air-dried for approximately 8 min before resuspension. The RNA pellet was resuspended in RNase-free water and incubated at 55 °C for 10 min to facilitate its dissolution. RNA concentration and purity were measured using a NanoDrop. RNA purity was confirmed by A260/A280 (1.8–2.0) and A260/A230 (2.0–2.2).

### 2.9. Real-Time qPCR

Purified and isolated RNA was reverse-transcribed into cDNA using the Verso cDNA Synthesis Kit (Thermo Fisher Scientific, Waltham, MA, USA) according to the manufacturer’s instructions. The primers (Thermo Fisher Scientific, Waltham, MA, USA) used included TNF-alpha (Mm00443258_m1), IL-1β (Mm00434228_m1), and IL-6 (Mm00446190_m1). Five microliters of the generated cDNA was used in qPCR, along with 10 μL of TaqMan 2× Universal PCR Master Mix (4364337, Thermo Fisher Scientific, Waltham, MA, USA), 2 μL of nuclease-free water, and 1 μL of the respective 20× TM primer. mRNA expression was quantified by normalizing to GAPDH (Mm99999915_g1), and the fold change was calculated. The specificity of the RT-qPCR was verified using non-template controls. Quantitative real-time PCR was performed using a QuantStudio™ 3 Real-Time PCR System (Thermo Fisher Scientific, Waltham, MA, USA).

### 2.10. Immunocytochemistry

BV2 microglial cells were seeded at a density of 1 × 10^5^ cells per well in a 12-well plate containing 1 mL of complete medium with coverslips and allowed to adhere for 12 h. The cells were then treated with EVs, followed by stimulation with LPS (100 ng/mL). Immunocytochemistry was performed following the treatments using standard protocols [16]. Briefly, the cells were gently washed with PBS, replaced with fresh PBS, and incubated on a rocker for 10–15 min at room temperature. Cells were fixed with 4% paraformaldehyde (PFA) in phosphate-buffered saline (PBS) for 15 min at RT. After fixation, the cells were washed three times with PBS for 5 min each on a rocking platform. Cells were then incubated with blocking buffer for 1 h at room temperature to minimize non-specific binding. The cells were permeabilized with 0.1% Triton X-100 (Thermo Fisher Scientific, Waltham, MA, USA). Next, the cells were incubated with IBA1 antibody (ab283342, Abcam, Waltham, MA, USA) overnight at 4 °C. The next day, the cells were incubated with 488-fluorophore-tagged secondary antibody (Alexa Fluor 488; A-11001, Invitrogen, Carlsbad, CA, USA) for 2 h at room temperature. Next, the cells were washed with PBS for 15 min at room temperature and mounted with DAPI (P36935, Invitrogen, Carlsbad, CA, USA). Imaging was performed using a Z1 inverted microscope (Carl Zeiss, Thornwood, NY, USA), and the analysis was performed using the AxioVision software (version 4.8.0.0; Carl Zeiss Microimaging GmbH, Jena, Germany). The mean fluorescence intensity was quantified using the ImageJ Launcher software (v1.4.3.67; NIH, Bethesda, MD, USA).

### 2.11. Statistical Analysis

All grouped data are presented as mean ± standard error of the mean (SEM). A *t*-test for two groups and a one-way analysis of variance (ANOVA) followed by Tukey’s post hoc test for multiple comparisons were used to assess statistical significance across groups using GraphPad Prism software (version 10), and a *p*-value < 0.05 was considered statistically significant.

## 3. Results

### 3.1. Characterization

**Figure 1:**
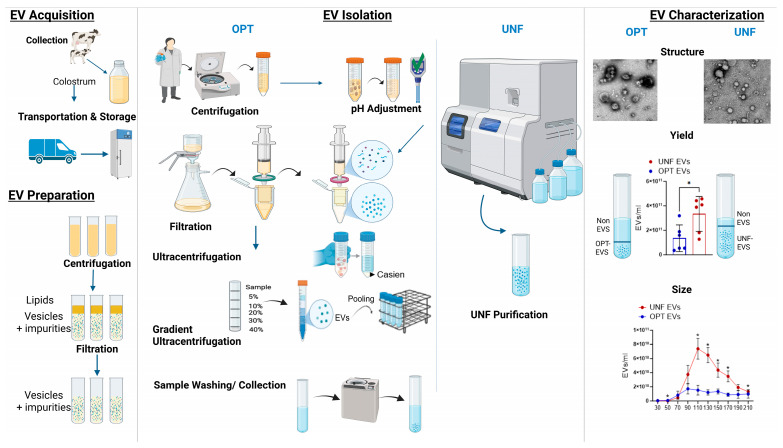
EV Isolation. Schematic representation of sample acquisition, the EV isolation and characterization using Opti-prep density gradient ultracentrifugation and Ultrasonic nanofiltration. * *p* < 0.05 versus Opti-prep EVs. Abbreviations: EV: Extracellular vesicles, OPT: OptiPrep-gradient EVs, UNF: Ultrasonic Nanofiltration EVs. Created in BioRender. Mehessen, N. (2026) https://BioRender.com/a7ahupu. (Accessed date on: 22 June 2026).

**Figure 2:**
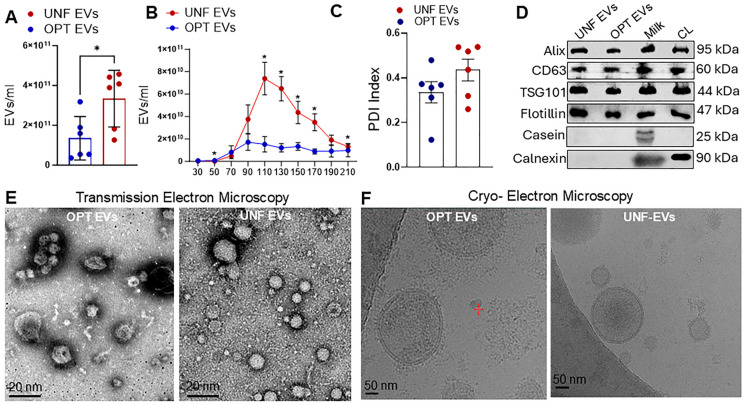
EV Characterization. (**A**) Concentration of EVs by NTA. (**B**) Particle size distribution by Nanoparticle Tracking Analysis. (**C**) Poly-Dispersity Index by DLS. (**D**) Representative Western blot images of exosome markers (Alix, CD63, TSG101, Flotilin), Negative EV marker (Calnexin), and Casein (milk protein). (**E**) Representative Transmission electron microscope images of isolated EVs. (**F**) Representative Cryo-EM images of isolated EVs. Data are expressed as Mean ± SEM. n = 6/group. * *p* < 0.05 versus Opti-prep EVs. Abbreviation: EV: Extracellular vesicles, OPT: OptiPrep-gradient EVs, UNF: Ultrasonic Nanofiltration EVs.

C-EVs were isolated using OPT and UNF (Figure 1). Isolated UNF EVs showed a significantly higher particle concentration than OPT EVs (Figure 2A). Nanoparticle tracking analysis showed that UNF EVs had a broader particle size distribution (50–210 nm) with a peak around 110 nm, whereas OPT EVs had lower particle counts across all size ranges, with a peak at 90 nm (Figure 2B). Dynamic Light Scattering Analysis showed a polydispersity index (PDI) of ~0.3–0.4 for both methods, indicating heterogeneous EV size (Figure 2B). Western blot analysis confirmed the presence of exosomal markers, including ALG-2 interacting protein X (Alix), cluster of differentiation (CD)—CD63, Tumor susceptibility gene 101 (TSG101), and flotillin, in EVs isolated by both methods (Figure 2D). In contrast, the ER marker calnexin (a negative EV marker) was absent in the isolated EV samples, indicating minimal contamination by cellular debris and intracellular organelles. To ensure that residual casein did not interfere with anti-inflammatory responses, Western blotting using a casein antibody (ab166596, Abcam, MA, USA) was performed, and the results showed that casein was absent in the isolated EVs. TEM revealed that EVs isolated by both methods had characteristic spherical and cup-shaped morphologies with intact membrane structures (Figure 2E). Cryo-EM further confirmed the presence of well-preserved lipid bilayer vesicles in all the samples (Figure 2F).

### 3.2. C-EV and Innate Immunity

**Figure 3:**
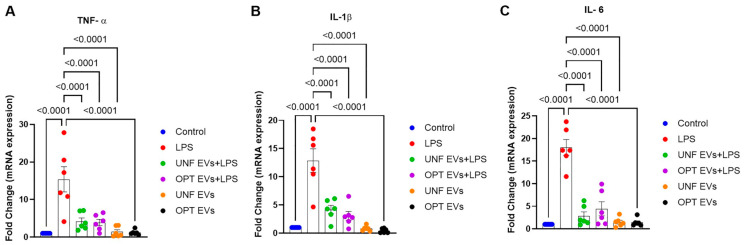
Role of UNF and OPT C-EVs to protect pro-inflammatory cytokines. mRNA expression of pro-inflammatory cytokines by qPCR analysis- (**A**) TNF-α, (**B**) IL-1β, (**C**) IL-6. Data are expressed as Mean ± SEM. n = 6/group. *p* < 0.05. Abbreviations: LPS: Lipopolysaccharide, EV: Extracellular vesicles, OPT: OptiPrep-gradient EVs, UNF: Ultrasonic Nanofiltration EVs.

Cytokines play a vital role in initiating innate inflammatory responses [19,20,21,22]. The microglial cell line (BV2 cells) was pretreated with C-EVs (from each of the two methods) for 22 h, followed by LPS (100 ng/mL) for 2 h to record the control of inflammatory responses. qPCR was performed to measure the expression of TNF-α, IL-1β, and IL-6 in each group. Quantitative PCR (qPCR) analysis revealed that LPS stimulation significantly upregulated (*p* < 0.0001) TNF-α, IL 1β, and IL6 expression compared to the control group. EV pre-treatment significantly (*p* < 0.0001) attenuated LPS-induced upregulation of IL-1β, IL-6, and TNF-α, indicating a pronounced anti-inflammatory effect of UNF and OPT colostrum EVs. Notably, EVs administered alone did not increase inflammatory gene expression, indicating that neither EV preparation elicited a pro-inflammatory effect under basal conditions (Figure 3A–C).

### 3.3. C-EVs Attenuate Inflammasome Activation

**Figure 4:**
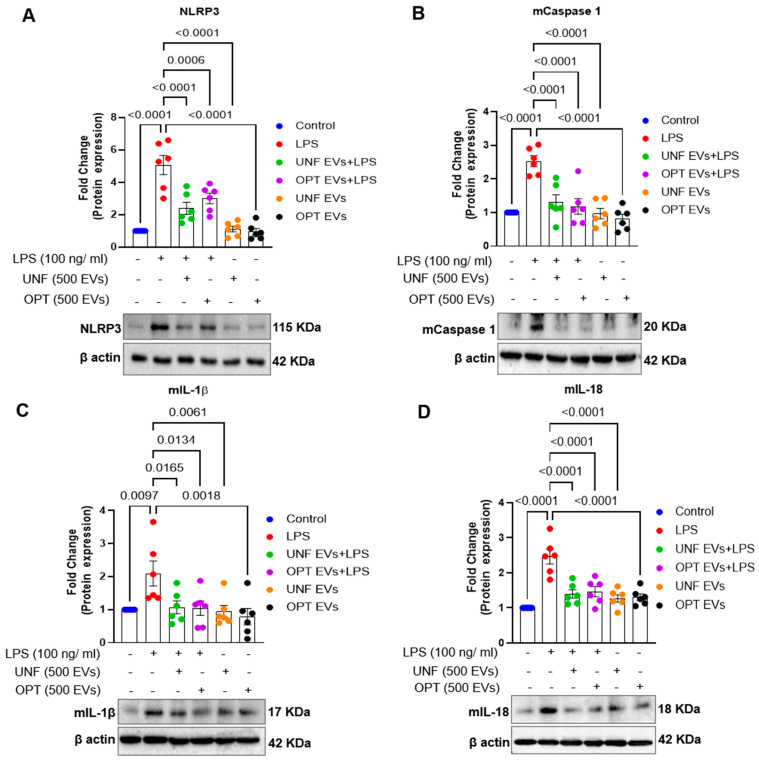
Role of UNF and conventional C-EVs in deactivating inflammasomes. Representative Western blot images showing the expression of NLRP3 (**A**), mature Caspase 1 (**B**), mature IL-1β (**C**), and mature IL-18 (**D**). Data are expressed as Mean ± SEM. n = 6/group. *p* < 0.05. Abbreviations: LPS: Lipopolysaccharide, EV: Extracellular vesicles, OPT: OptiPrep-gradient EVs, UNF: Ultrasonic Nanofiltration EVs.

Next, C-EVs were assessed using Western blotting to determine the modulation of inflammasome expression. LPS stimulation significantly upregulated NLRP3 expression compared to that in the control group (*p* < 0.0001). EVs isolated using either method significantly suppressed the LPS-induced increase in NLRP3 expression (*p* < 0.0001) (Figure 4A). Similar results were observed for the other components of the NLRP3-signaling pathway. As shown in Figure 4B–D, LPS induced a significant increase (*p* < 0.0001) in the expression of mature caspase-1, lL1β, and IL18, whereas C-EV pre-treatment suppressed (*p* < 0.0001) LPS-induced mature caspase-1, lL1β, and IL18. In contrast, treatment with EVs alone did not significantly alter NLRP3 or mature forms of caspase-1, IL-1β, or IL-18 expression compared to the control group, suggesting that EVs are not cytotoxic or pro-inflammatory.

### 3.4. C-EV Controls Microglial Activation

**Figure 5:**
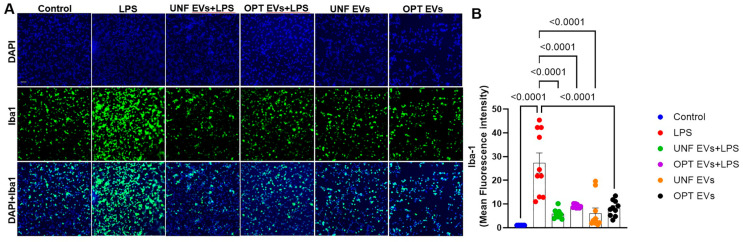
Role of UNF and OPT EVs on microglial activation: Representative immunocytochemistry images of Iba1-stained BV2 cells (**A**) and quantification of mean fluorescence intensity (**B**). Data are expressed as Mean ± SEM. n = 6/group. *p* < 0.05. Abbreviations: LPS: Lipopolysaccharide, EV: Extracellular vesicles, OPT: OptiPrep-gradient EVs, UNF: Ultrasonic Nanofiltration EVs.

Next, to assess the comparative role of C-EVs isolated by both methods in microglial activation, we performed immunocytochemistry for microglial Iba1 in each group. As shown in Figure 5A, LPS induced increased (*p* < 0.0001) Iba1 expression, demonstrating microglial activation compared to the control, while pretreatment with EVs significantly suppressed (*p* < 0.0001) LPS-induced microglial activation. Furthermore, quantification data demonstrated that the mean fluorescence intensity of Iba-1 was significantly decreased (*p* < 0.0001) upon pre-treatment with either isolation technique compared to LPS alone. In addition, treatment with EVs did not induce microglial activation (Figure 5B).

**Figure 6:**
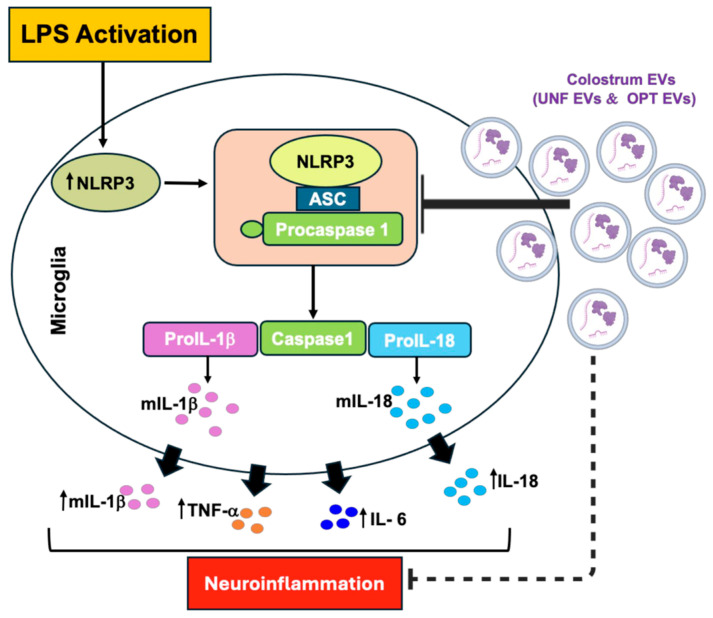
Summary of the anti-inflammatory effects of EVs derived from Milk Colostrum on LPS-exposed BV2 cells. Abbreviations: EV: Extracellular vesicles, ASC: apoptosis-associated speck-like protein containing a CARD, OPT: OptiPrep-gradient EVs, UNF: Ultrasonic Nanofiltration EVs. Created in BioRender. Akter, S. (2026) https://BioRender.com/mzamfn9 (Accessed date on: 22 June 2026).

LPS stimulation activates the NF-κB signaling pathway and the formation of the NLRP3-ASC inflammasome complex in microglial cells. This activation increases the transcription of pro-inflammatory precursors, including pro-caspase-1, pro-IL-1β, and pro-IL-18, which are subsequently cleaved by caspase-1 into their mature active forms. Elevated levels of inflammatory cytokines, including IL-1β, IL-18, TNF-α, and IL-6, were observed, contributing to neuroinflammation. Pre-treatment with colostrum-derived extracellular vesicles (EVs), isolated using both methods, attenuated these inflammatory responses (Figure 6).

## 4. Discussion

Ultrasonic nanofiltration-based EV isolation has significant advantages. This was recorded in terms of time, recovery, and preservation of functional anti-inflammatory EV activities [23]. While ultracentrifugation is similarly capable of eliciting functional responses, concerns remain regarding the capture of pure exosomes and the removal of contaminants that can trigger adverse immune responses [24]. The reductions in isolation times relative to the conventional 30 h protocols led to higher yields and reduced potential for batch-to-batch variance, which can be critical for clinical translational studies. Future studies should assess protein removal and free-pure recovery. Perhaps the most notable advance is label-free exosome recovery. There was no reliance on antibodies, which could compromise the biological integrity of EVs [25]. The absence of particle aggregation during the extraction procedure and the potential to recover functionally intact particles highlighted significant advantages over more commonly used techniques [24].

EVs have notable therapeutic potential in various [26]. Morphologically, they are characterized by a lipid bilayer membrane [27] and are reflective of the cell sources from which they are produced [28]. EVs range in size from approximately 30 nm to 10 µm [29] and are categorized into subtypes. These include exomeres, exosomes, small EVs, and apoptotic bodies [30]. EVs are released by all living cells and can be isolated from a wide range of biological fluids, including blood, saliva, urine, cerebrospinal fluid, breast milk, and bovine milk [31]. However, their biological characteristics and functions largely depend on their biogenesis pathways [12]. Among EV sources, milk-derived bovine EVs have attracted interest because of their biocompatibility, low immunogenicity, and disease-fighting therapeutic potential [32]. EVs are now regarded as critical components in maintaining cellular homeostasis under normal physiological conditions owing to the unique characteristics and functions of their bioactive cargo [33]. They act as intercellular communication vehicles, mediating paracrine, endocrine, and autocrine signaling. This is maintained either by cell surface interactions without cargo release, by membrane fusion with target cells, or endocytosis. The EV cargo is released into the cytoplasm or endosomal compartment, allowing for endosomal escape to facilitate a biological effect [34,35,36]. The variety of biological effects of EVs is broad [37,38], including modulation of pro- and anti-inflammatory immune responses [39], tissue regeneration and repair [39], and control of neurological function, particularly synaptic plasticity, myelination, and neuroprotection [11,40,41,42]. EVs have demonstrated therapeutic potential in various neurodegenerative diseases [43,44], including PD [11,45,46,47] and AD [32,48,49,50,51]. Functionally, bovine C-EVs significantly suppress the expression of apoptosis-related genes, including Bax, p53, and caspase-3, as well as pro-inflammatory cytokines, such as TNF-α, IL-6, and IL-1β, in intestinal epithelial cells [52]. In contrast, mature milk-derived EVs showed limited effects on apoptosis-related genes and primarily reduced the expression of inflammatory mediators, including TNF-α and IL-6, in intestinal epithelial cells [52]. In contrast, C-EVs accelerate wound healing by promoting fibroblast proliferation and migration, enhancing angiogenesis, regulating extracellular matrix remodeling, and suppressing inflammation [53]. In addition, C-EVs exhibit antimicrobial, anti-inflammatory, and immunomodulatory activities in neonatal calf diarrhea models by reducing bacterial adherence and modulating immune-related gene expression [54]. C-EVs also protect intestinal epithelial cells from LPS-induced injury by improving barrier integrity, enhancing cell proliferation, and reducing apoptosis and inflammatory responses, exerting stronger protective effects than mature EVs [52]. Recently, bovine colostrum EVs have been reported to alleviate atopic dermatitis by modulating the gut–skin axis, restoring gut microbiota balance, regulating immune responses, and improving intestinal metabolite profiles [55]. Collectively, these findings highlight the therapeutic and regenerative potential of bovine colostrum-derived EVs as promising natural nanotherapeutics for inflammatory and immune-related disorders.

Our previous study demonstrated that bovine C-EVs have anti-inflammatory and neuroprotective effects in a methyl-4-phenyl-1,2,3,6-tetrahydropyridine (MPTP) model of PD [11]. In a recent study, we demonstrated that C-EV treatment rescued dopaminergic neurons, reduced the number of reactive microglia, and diminished inflammasome cascade expression, thereby decreasing neuroinflammation. Based on this background, we employed an upgraded UNF to isolate C-EVs using exosome detection by UNF, which employs an automated dual-membrane nanofiltration system integrated with a periodic negative NPO and double-coupled HO (Exodus) [15], and compared it with OPT-isolated EVs for structural and functional integrity.

We accept that, to date, the clinical adoption of C-EVs remains restricted [44]. EVs have generally been avoided in human treatments because of production challenges, safety concerns, and poor targeted delivery [56]. One key limitation that has stopped widespread clinical use includes, but is not limited to, EV isolation and standardization. It has been difficult to separate pure, uniform preparations of EVs from other cellular materials containing proteins and lipids [57]. These challenges, among others, must be overcome to guarantee that each batch of vesicles isolated from diverse materials, including cells and body fluids, meets the requirements of any regulatory human drug. Isolating pure and uniform EVs is technically challenging. Recurrent concerns stem from the fact that EVs are commonly derived from living cells; thus, there remains a risk of viral, endotoxic, and other cellular contaminants [58]. Due to these contamination risks, EVs can trigger adverse immune reactions or promote cancer. There is also a need to overcome poor tissue targeting. When EVs are injected systemically, most are rapidly cleared by the liver and spleen before they reach the target tissues of interest, which requires high dosing and carries an additional potential for long-term toxicities. We also understand that no approved EV-based therapies currently exist, which means that such therapies remain highly experimental. Available preclinical studies have demonstrated that bovine C-EVs exhibit favorable biocompatibility and low immunogenicity. However, their long-term safety, biodistribution, pharmacokinetics, and potential off-target effects require further investigation before clinical application. C-EVs bypass immune clearance and prevent unwanted inflammatory or toxic responses when circulating in the body [11]. Second, C-EV payloads, which consist of beneficial microRNAs, proteins, and lipids, act as gene expression modulators rather than toxins, allowing them to selectively alter cellular machinery without inducing notable cytotoxicity [11,55]. Third, C-EVs are engineered to withstand harsh environments, such as the gastrointestinal tract, enabling them to safely cross biological barriers without triggering systemic toxicity or off-target adverse effects [59,60]. C-EVs naturally carry high concentrations of growth- and immune-related proteins that actively suppress inflammation and reduce oxidative stress, thereby protecting the body from unintended toxicities.

To evaluate the feasibility, efficiency, and scalability of ultrasonic nanofiltration for isolating C-EVs, we compared it with our previously optimized EV isolation gradient method. Characterization of C-EVs confirmed successful isolation using both methods, with conventional ultracentrifugation requiring an extended time, whereas UNF isolation was completed in just a few hours. The yield of UNF C-EV was 2–3-fold higher than that of OPT-EV. Western blot analysis demonstrated the presence of canonical exosomal markers (ALIX, CD63, TSG101, Flotillin) in the isolated exosomes, while the absence of calnexin confirmed minimal contamination from intracellular organelles. Furthermore, the absence of casein further validated the purity of the EV preparations, indicating the effective removal of milk protein contaminants. Morphological analysis by EM confirmed the characteristic cup-shaped, membrane-bound vesicular structure with intact lipid bilayers, supporting the structural integrity of EVs isolated using both methods. Functionally, both EV preparations significantly suppressed LPS-induced neuroinflammatory responses by modulating key pathways involved in microglial activation and inflammasome signaling. Notably, NLRP3 drives chronic inflammation by activating caspase-1, which processes IL-1β/IL-18 and triggers pyroptotic cell death [61,62]. The induction of NLRP3, along with the activation of downstream signaling molecules, including cleaved caspase-1, mature IL-1β, and IL-18. Both UNF and OPT EVs significantly suppressed NLRP3 inflammasome activation. The reduction in caspase-1 activation and downstream cytokine maturation suggests that C-EVs act at an upstream regulatory level, affecting inflammasome assembly and NF-ƙB-dependent priming signals [63]. The anti-inflammatory role of C-EVs was demonstrated by pre-treatment with C-EVs, which robustly attenuated the inflammatory response. The results demonstrated a broad anti-inflammatory response in C-EVs. Microglia support neural homeostasis [64]. Chronic activation drives the release of pro-inflammatory cytokines, disrupting neuron–microglia communication, impairing synaptic structure and plasticity, promoting excitotoxicity, and promoting neurodegeneration [65].

Overall, the combined results of EV characterization, cytokine profiling, inflammasome signaling, and Iba1 immunostaining provide strong and consistent evidence that bovine colostrum-derived EVs possess potent immunomodulatory properties. Their ability to suppress both microglial activation and NLRP3 inflammasome signaling highlights the anti-inflammatory activities for the treatment of neuroinflammatory and neurodegenerative diseases. Given the central role of chronic microglial activation [65] these findings suggest that C-EVs may represent a promising strategy for mitigating a broad range of diseases both inside and outside the nervous system. Furthermore, UNF Exodus EVs yielded higher recovery and reduced isolation time. These data affirm that EVs can be developed as an anti-inflammatory therapeutic in clinical settings.

Our study limitations include functional primary human cell analyses, as the inflammatory profiles were completed in LPS-stimulated BV2 microglial cells. This cell model represents a simplified in vitro system for studying microglial inflammation. Although BV2 cells are widely used to investigate cell-specific signaling pathways, they do not fully recapitulate the complexity of primary microglia or the multicellular environment within the central nervous system. Furthermore, LPS-induced inflammation only reflects one aspect of neurodegenerative pathology. It does not reproduce progressive neuronal loss, protein aggregation, or cellular interactions seen in common neurodegenerative disorders, including Alzheimer’s and Parkinson’s Diseases. Therefore, the anti-inflammatory effects observed in this study only reflect the cell-linked immunomodulatory activities. Future studies using primary microglia, co-culture systems, and more clinically relevant in vivo disease models will affirm these findings and translate them into broader neuroprotective activities.

## 5. Conclusions

This study demonstrates that bovine C-EVs can be isolated using the ultrasonic nanofiltration platform in substantially less time and with higher recovery than the optimized density-gradient ultracentrifugation method. EVs obtained by both approaches preserved vesicle integrity and retained the ability to attenuate inflammatory responses in LPS-stimulated BV2 microglial cells. Overall, these findings suggest that the Exodus UNF-based isolation method is a rapid, scalable, safe, and efficient platform for producing biologically active C-EVs with therapeutic potential. However, the biological effects reported here are limited to a single in vitro neuroinflammatory model used in this study. Future studies will be conducted to assess the cargoes in C-EVs (immunoglobulins, antibodies and miRNAs) that potentiate anti-inflammatory effects. Immunoglobulins (IgG and IgA, in particular) with EVs can form a unique immune synergy. Colostrum-derived secretory IgA is a major component of early mucosal immunity, forming a protective barrier over the intestinal epithelium and thereby reducing exposure to environmental antigens and pathogens during the critical neonatal period [66,67]. Notably, population-specific differences in neonatal IgA levels have been reported, highlighting the influence of maternal, environmental, and geographical factors on early-life immune protection [68]. In addition, emerging evidence suggests that extracellular vesicles may interact with or transport immune mediators, including immunoglobulins, potentially contributing to maternal-to-infant immune communication [68,69]. In addition to reports demonstrating functional antibody expression on extracellular vesicles, immunoglobulin heavy and light chains have also been detected in serum-derived exosomes from animal models and human biological fluids, suggesting that EVs may associate with or transport immunoglobulins in vivo [70]. These findings suggest that EVs contribute to antibody-mediated immune communication, although the underlying mechanisms and biological significance warrant further investigation. EV can form a non-circulatory system for antibody delivery. Such studies can assess long-term functions and evaluate EV stability in lyophilized formulations. In addition, the storage conditions, biodistribution, pharmacokinetics, safety, and efficacy of these nanoparticles need to be investigated in future studies to enable their clinical translation.

## Data Availability

The original contributions presented in this study are included in the article. Further inquiries can be directed to the corresponding authors.
